# Comparison of echocardiographic linear dimensions for male and female child and adolescent athletes with published pediatric normative data

**DOI:** 10.1371/journal.pone.0205459

**Published:** 2018-10-11

**Authors:** Hubert Krysztofiak, Łukasz A. Małek, Marcel Młyńczak, Andrzej Folga, Wojciech Braksator

**Affiliations:** 1 Mossakowski Medical Research Centre, Polish Academy of Sciences, Warsaw, Poland; 2 National Centre for Sports Medicine, Warsaw, Poland; 3 Faculty of Rehabilitation, Józef Piłsudski University of Physical Education, Warsaw, Poland; 4 Institute of Metrology and Biomedical Engineering, Faculty of Mechatronics, Warsaw University of Technology, Warsaw Poland; 5 Departament of Sports Cardiology and Noninvasive Cardiovascular Imaging, 2nd Medical Faculty, Medical University of Warsaw, Warsaw, Poland; Universita degli Studi di Roma La Sapienza, ITALY

## Abstract

**Background:**

Application of normative data for echocardiographic measurements to children practicing sports may lead to false positive findings. The aim of the study was to define the normative data of basic echocardiographic measurements for this group and to compare them to the previously published normative data for the pediatric population.

**Methods:**

Parasternal long-axis 2D-guided echocardiographic measurements were obtained from a group of 791 child athletes (ages 5–18 years). According to the methodology presented previously by Pettersen et al. (2008), the regression equations for basic cardiac dimensions against body surface area were derived and individual Z-scores values were computed, using both Pettersen’s equations and newly derived ones.

**Results:**

Z-scores computed based on Pettersen’s equations were found to differ significantly from those based on the new equations, for all the analyzed parameters (p<0.001). In agreement analysis, the most pronounced differences were found for the left atrium, interventricular septum and the left ventricular posterior wall. However, in most cases, the indications of abnormality were concordant (91.8%–97.6%); except for the left atrium, where there were 30.8% discordant results.

**Conclusion:**

The study presents normative data for basic echocardiographic cardiac measurements for children of both sexes practicing varying sporting disciplines and compares them with general pediatric population. Mean values of cardiac dimensions are higher in young athletes, particularly in relation to the left atrium and left ventricular muscle thickness. In most cases, the upper limit of normality observed in the young athletes is confined within the upper limit of the general pediatric population.

## Introduction

An echocardiogram is not a mandatory element of pre-participation evaluation (PPE) of athletes, but some children and adolescents engaging in sports may undergo this examination is case of suspected abnormalities found during periodical assessment, such as suspicious family history, clinical symptoms, heart murmurs, or atypical electrocardiogram changes [1]. Usually, basic left and right ventricular and left atrial cavity size parameters obtained during transthoracic echocardiography are compared to normal values for healthy children not participating in regular athletic activities [2–4]. However, it has been well documented over the past few decades that regular physical activity leads to physiological cardiac adaptation called “athlete’s heart,” consisting mainly of enlarged heart chambers, and increased left ventricular (LV) muscle thickness [1,5–8]. It has since been demonstrated that the remodeling depends mainly on sport intensity and cumulative duration of training, with the ratio of LV mass to LV size preserved regardless of sport [9] Although adaptive changes are most pronounced in elite adult athletes, some degree of “pediatric athlete’s heart” has also been recognized recently [10–13] Therefore, application of normative data for the general pediatric population to child and adolescent athletes may lead to false positive findings of enlarged heart structures and LV muscle thickness. As a consequence this may lead to unnecessary stress for the child and its parents, cause unjustified further testing to exclude pathology or even lead to disqualification of young athlete from sport. For this reason, comparison of normative data dedicated to this group with general population seems warranted.

Accordingly, the aim of this study was to define normative data for basic linear echocardiographic measurements for children and adolescents practicing sports and to compare them to the published normative data for the pediatric population.

## Methods

### Study group

This retrospective study assessed healthy white children and adolescents (327 girls and 464 boys, 5–18 years old, mean age 13±5 years) engaged in regular athletic training at the local or national level (mainly soccer, track and field, basketball, swimming and martial arts). Detailed characteristics of the study group, including age, weight, height, body surface (BSA), and training volume are presented in Table 1.

**Table 1:** Study group characteristics.

	Whole group	Age categories				
		5–6 years	7–9 years	10–12 years	13–15 years	16–18 years
Number of subjects	791	20	180	250	230	111
Male sex	464–59%	12–60%	105–58%	126–50%	135–59%	86–77%
Age [years]	12 (5)	-	-	-	-	-
Height [m]	156 (30)	122 (11)	135 (9)	151 (13)	168 (12)	179 (11)
Body mass [kg]	44.30 (26.05)	21.8 (6.0)	29.4 (7.7)	39.4 (11.4)	56.0 (14.8)	68.5 (12.7)
BSA [m^2^]*	1.39 (0.54)	0.85 (0.15)	1.05 (0.17)	1.27 (0.23)	1.61 (0.27)	1.84 (0.20)
Training volume [min]**	270 (180)	180 (135)	180 (120)	180 (180)	360 (180)	540 (180)
LVD	4.4 (0.8)	3.7 (0.3)	3.9 (0.5)	4.2 (0.5)	4.7 (0.4)	5.0 (0.6)
RVOT PLAX	1.9 (0.6)	1.5 (0.3)	1.7 (0.4)	1.8 (0.5)	2.0 (0.4)	2.3 (0.7)
IVS	0.8 (0.2)	0.6 (0.1)	0.7 (0.1)	0.8 (0.1)	0.8 (0.1)	0.9 (0.1)
PWD	0.8 (0.2)	0.6 (0.1)	0.7 (0.1)	0.7 (0.1)	0.8 (0.1)	0.9 (0.1)
LA	3.0 (0.6)	2.5 (0.4)	2.7 (0.3)	2.9 (0.4)	3.2 (0.4)	3.4 (0.4)
AO	2.3 (0.6)	1.8 (0.3)	2.0 (0.4)	2.2 (0.4)	2.4 (0.5)	2.6 (0.6)

All unit-bearing values are represented as “median (interquartile range)”

* BSA was calculated based on the Haycock formula^14^: *BSA* (*m*^2^) = 0.024265 ∙ *Height*(*cm*)^0.3964^ ∙ *Weight*(*kg*)^0.5378^

** The training volume determines the level of involvement in a sport and was estimated as the product of the average number of training sessions per week and the average duration of a single training session.

All study participants had undergone transthoracic echocardiography as a part of periodic PPE due to innocent heart murmurs or suspicion of abnormal electrocardiographic findings. The studies were performed at the National Centre for Sports Medicine between 2013 and 2017. Children thus found to have significant acquired or congenital heart diseases affecting normal heart size and hemodynamics were excluded.

### Echocardiography

Echocardiograms were performed by two experienced sonographers with using a commercially available ultrasound scanner (Toshiba Aplio 400, Toshiba Medical Systems Europe, Zoetermeer, the Netherlands), according to recent guidelines. All measurements were taken in 2-dimensional parasternal long axis view (PLAX) and included basic linear cardiac dimensions: interventricular septum diameter at end diastole (IVS), left ventricular posterior wall diameter at end diastole (PWD), left ventricular diameter at end diastole (LVD) and right ventricular diameter at end diastole (RVOT PLAX), aortic sinus diameter at end systole (AO) and left atrial diameter at end systole (LA). All measurements were taken from inner edge to inner edge and reported to within 1 mm. Persons exhibiting ambiguous results of any measurement were excluded from the initial study group. Body surface area (BSA) was calculated according to the Haycock formula [14].

### Ethical considerations

The study, including the consent procedure, was approved by the Ethics Committee of Warsaw Medical University (permission AKBE/75/17). As the study was retrospective neither written nor verbal consent for this particular study was obtained, but each subject had signed the informed consent form for the routine medical monitoring, including the statement of agreement for the use of the results for the scientific purposes.

### Statistical methods

For preparation of normative data and for comparison, we used the methodology described in the manuscript reporting the most frequently used normative echocardiographic cardiac dimensions data for a general population of children [4]. In brief, each measurement was transformed by computing its natural logarithm (*y = ln[measurement]*). Each transformed measurement was entered into a nonlinear (polynomial) regression model as the dependent variable, and BSA, BSA^2^, and BSA^3^ as the predictors (independent variables), yielding an equation of the form:

$$Expected\;y=\beta_{0}+\beta_{1}∙BSA+\beta_{2}∙{BSA}^{2}+\beta_{3}∙{BSA}^{3}$$

Observations for which the studentized error residuals of the linear model relating cardiac parameter and BSA were above +3 or below -3 were excluded [4]. As a result, the number of measurements for each cardiac dimensions used to fit the model was different, ranging from 782 to 789. For each cardiac dimension, we assessed the statistical significance of every coefficient (β0 to β3, using T statistics). Once the regression coefficients were obtained, the equation was transformed back to the measurement’s original scale by exponentiation of *expected y*.

As many coefficients were statistically insignificant, we decided to evaluate and adjust the formulas recursively, beginning from the highest power, in order to produce an optimal model. We were removing iteratively those coefficients, which were not significant. Also, nonsignificant intercept parameters were excluded if appropriate. The coefficients of the models, which appeared optimal in terms of utilized input variables are stored in supplementary materials (S2 File), along with specific p-values (the differences in curves’ shapes between original and optimal models are very slight).

Next, Z-scores were calculated separately for all the dimensions, based on the equation:

$$z=\frac{\ln\left(measured\;dimension\right)-expected\;y}{\sqrt{MSE}}$$

using our fitted models and those proposed by Pettersen et al [4]. In order to compare the differences of Z-scores of each cardiac dimension, obtained for all 791 participants, using model coefficients estimated for our group and for the general population, a paired t-test was used.

This was followed by estimation of agreement between model curves and Z-scores. For the first purpose, we placed on a single graph the mean and ±1SD, ±2SD, and ±3SD curves from both models, for the range of BSA values between 0.3 and 2.1 m^2^. For the second purpose, the mean and critical differences (1.96 SD) determined from a Bland-Altman plot were analyzed. In order to analyze the concordant and discordant indications of abnormal cardiac dimensions according to the newly created and previously published normative data, we constructed scatterplots of the Z-score values obtained using the two different models with normal boundaries (+1.65 Z-score) (per Pettersen et al. [4]). We estimated the parameters describing, the percentage of all results in the discordant area.

All calculations used R software (version 3.4.2, “Short Summer”; R Foundation, Vienna, Austria, http://www.r-project.org), along with external packages. For all statistical tests, a significance level of α = 0.05 was applied.

## Results

### Reference values for children practicing sports

**Fig 1:**
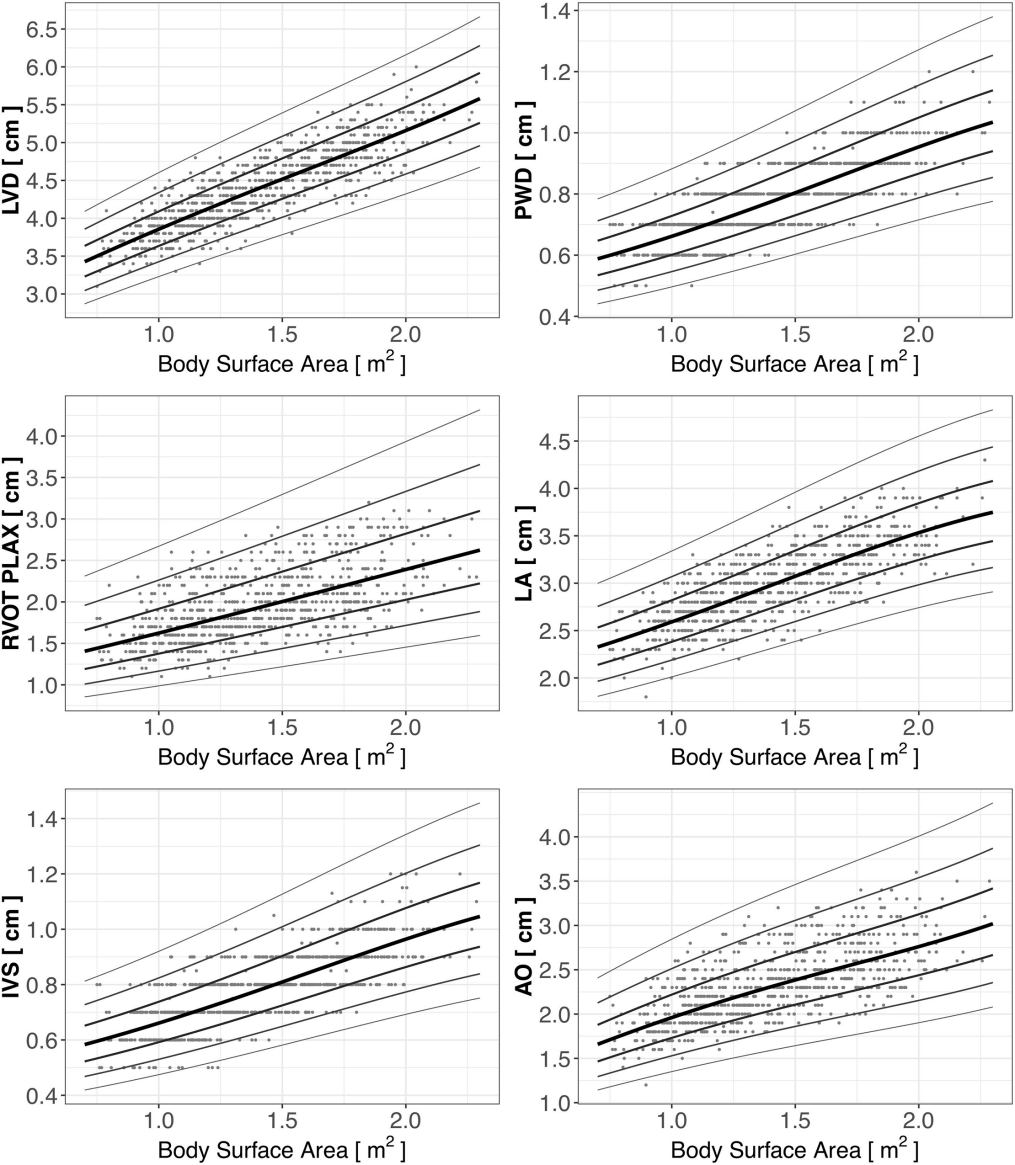
Scatter plots of cardiac dimensions versus BSA in children practicing sports, with estimated reference curves.

Reference scatter plots of the analyzed cardiac dimensions in the analyzed group of children practicing sports are presented in Fig 1. The black line represents the Z-score = 0, which is the estimated mean, whereas other lines represent Z-score values of ±1, 2, and 3, corresponding to 1, 2, and 3 standard deviations. The parameters used in calculation of the Z-scores are presented in Table 2.

**Table 2:** Coefficients for regression equations relating echocardiographic measurements and body surface area, mean squared error, and adjusted coefficient of determination.

Parameter	N	Int (β0)	p	BSA (β1)	p	BSA^2^ (β2)	p	BSA^3^ (β_3)_	p	MSE	R^2^
LVD	787	0.8529	<0.001	0.6692	<0.05	-0.2075	ns	0.0349	ns	0.00349	0.732
RVOT PLAX	788	-0.0623	ns	0.6457	ns	-0.1076	ns	0.0091	ns	0.02752	0.379
IVS	788	-0.8055	<0.01	0.3339	ns	0.0898	ns	-0.0323	ns	0.01221	0.566
PWD	782	-0.7767	<0.001	0.2984	ns	0.0973	ns	-0.0321	ns	0.00922	0.615
LA	785	0.6085	<0.01	0.3005	ns	0.0736	ns	-0.0302	ns	0.00714	0.602
AO	789	-0.1132	ns	1.1712	ns	-0.4667	ns	0.0816	ns	0.01548	0.468

AO–aortic sinus diameter at end systole, BSA—body surface area, Int–intercept, IVS–interventricular septum diameter at end diastole, LA–left atrial diameter at end systole, LVD–left ventricular diameter at end diastole, MSE—mean squared error, PWD—left ventricular posterior wall diameter at end diastole, RVOT PLAX—right ventricular diameter at end diastole in parasternal long axis

$$Expected\;y=\beta_{0}+\beta_{1}∙BSA+\beta_{2}∙{BSA}^{2}+\beta_{3}∙{BSA}^{3}$$

$$z=\frac{\ln\left(measured\;dimension\right)-expected\;y}{\sqrt{MSE}}$$

Based on the regression coefficients presented in Table 2 a precise Z-score can be calculated for individual child. Let's assume as an example a 13 year old boy, with a height of 157 cm and a weight of 48 kg which gives BSA = 1.44. His LVD measured during echocardiographic examination is 4.7 cm. To calculate the Z-score of this boy's LVD, in the first step one has to obtain the expected value of natural logarithm of LVD for BSA = 1.44 inserting corresponding regression coefficients from Table 2 into the first equation presented above in the section "Statistical methods":

$$Expected\;\ln\left(LVD\right)=0.8529+0.6692∙1.44-0.2075∙{\left(1.44\right)}^{2}+0.0349∙{\left(1.44\right)}^{3}=1.4905$$

In the next step, the second equation presented above should be used. The difference between natural logarithm of boy's LVD and the expected value of natural logarithm of LVD for BSA = 1.44 should be divided by the square root of the mean squared error (MSE) from Table 2:

$$z=\frac{\ln\left(4.7\right)-1.4905}{\sqrt{0.00349}}=0.9659$$

### Comparison of our reference values with reference values for the general population

**Fig 2:**
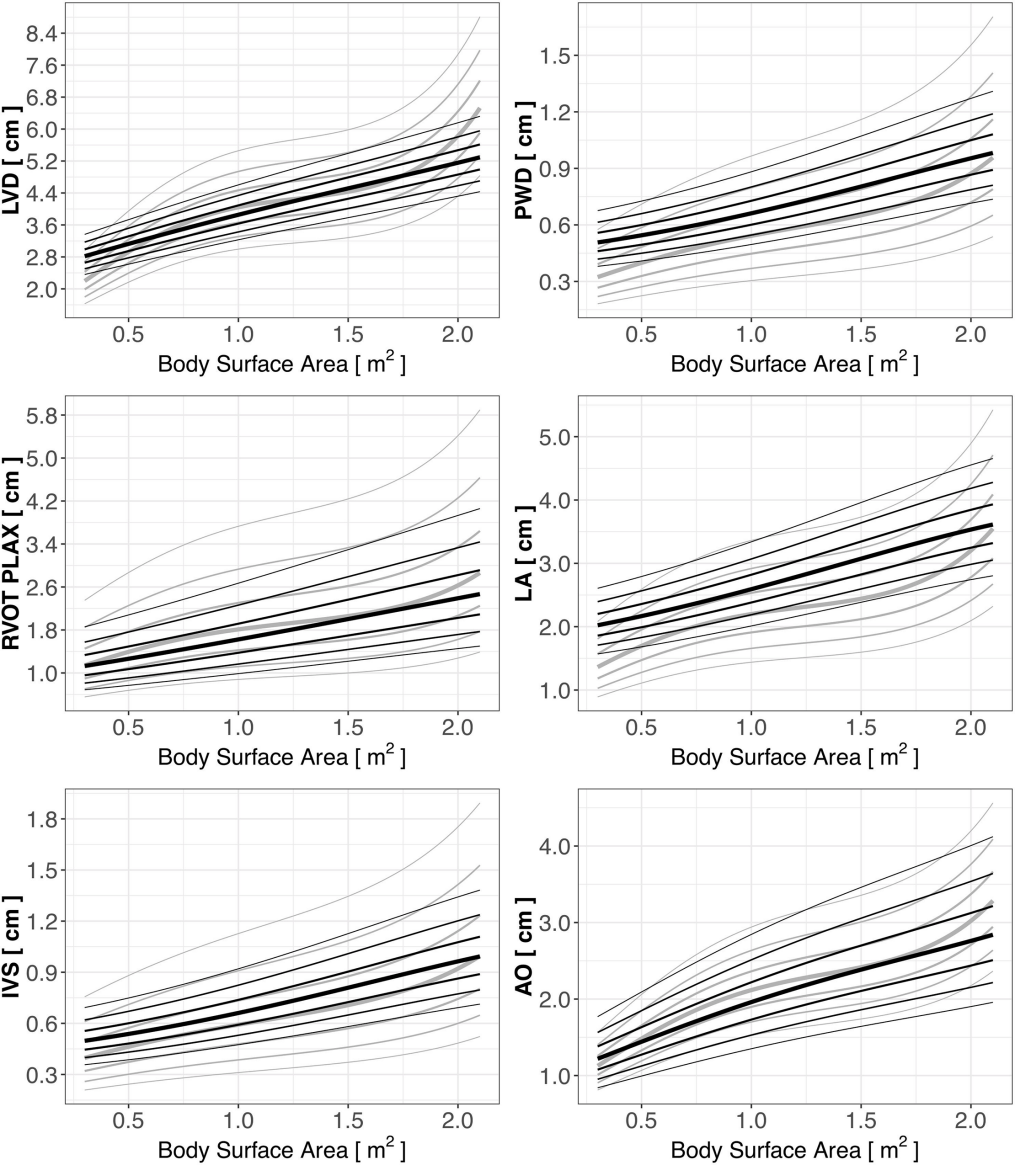
Z-scores of cardiac dimensions in children practicing sport (black lines) and in the general population of children (light gray lines) [4].

Next, we superimposed the Z-score lines created for children practicing sports (black lines) on the Z-score lines of cardiac dimensions for the general pediatric population (light gray) published previously by Pettersen et al [4] (Fig 2). The differences between the means of the Z-score values (Z-score = 0, thick black and grey lines on Fig 2) computed based on the Pettersen equations and those computed based on the new equations were statistically significant for all the analyzed parameters, as determined with the paired t-test (p<0.001). However, the most pronounced differences were found for LA, IVS, and PWD, with only modest differences for other parameters. As shown on Fig 2 the boundaries of cardiac dimensions in young athletes (thin black lines) in general confined within those for general population (thin grey lines) except LA where higher values in athletes were observed. The Bland-Altman analysis of agreement between the Z-score values for all the cardiac dimensions is presented in Table 3.

**Table 3:** Mean and critical differences between child athletes and children in general for each analyzed cardiac dimension^4^.

Parameter	Mean	Critical difference
LVD	0.2344898	1.2690
RVOT PLAX	0.2691593	0.6904
IVS	-0.5256694	1.0017
PWD	-0.9716611	1.1122
LA	-1.3528533	1.0315
AO	0.4141895	0.6564

Parameters as in Table 2.

**Fig 3:**
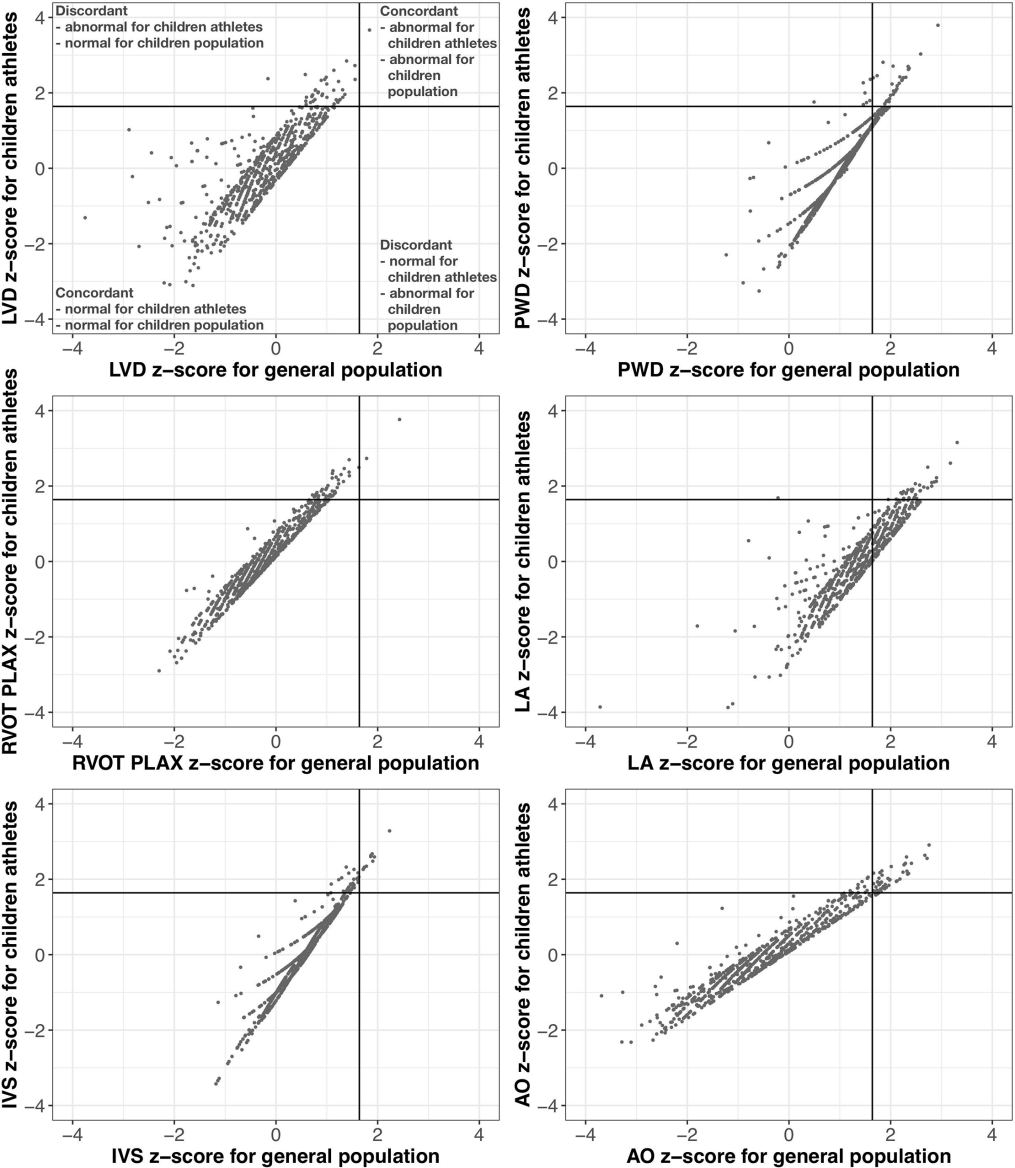
Scatterplots of the Z-score values showing concordant and discordant indications for abnormal cardiac dimensions (+1.65 Z-score) values according to Z-scores obtained for child athletes and a general population of children [4].

Finally, we performed scatterplots of the Z-score values obtained using the two different models with normal boundaries (+1.65 Z-score) to detect the percentage of concordant and discordant indications of abnormal cardiac dimension values according to Z-scores computed based on the Pettersen equations and on the new equations (Fig 3). The indications were concordant in most cases (95.2% for LVD, 94.7% for RVOT PLAX, 96.2% for IVS, 91.8% for PWD, 97.6% for AO), except for LA, where 30.8% of results were discordant. In this case, as well as in the case of PWD, the discordance was caused mainly by instances where Pettersen’s model indicated abnormality while ours indicated normality, suggesting physiological adaptation of those parameters to training. Interestingly, about 95% of Z-score values calculated using Pettersen’s coefficients were greater than 0 (p<0.001). For other cardiac dimensions, the discordance was due to the opposite–normality according to our model and abnormality according to Pettersen’s model, which may signify narrowing of the Z-scores in child athletes, as discussed later.

## Discussion

This study presents normative data on basic echocardiographic linear measurements for male and female children and adolescents practicing varying sporting disciplines and compares them with previously published normative data for general pediatric population^4^. To out best knowledge there are only one recently published Z-score values for left heart echocardiographic dimensions and mass in male peri-pubertal athletes [15].

### Cardiac adaptation to exercise in children

Cardiac adaptation to regular exercise is a well-described phenomenon most pronounced in elite adult athletes [1,5–8] but also present to a lesser extent in children [10–13] By comparing the Z-scores of basic echocardiographic measurements computed based on the most frequently used normative data for children and adolescents and those computed based on the new data specific to child and adolescent athletes, we were able to demonstrate that cardiac dimensions in young athletes are significantly different than in the general population of children. In particular, we found that children practicing sports have larger left atria and thicker left ventricular muscles. Smaller differences were observed for other parameters such as left and right ventricular diameters and aortic sinus diameters.

Our findings are in line with previously published studies on children. Increase in LV muscle thickness in response to training was found by Krustrup et al. and Sharma et al. using echocardiography and by our group using cardiac magnetic resonance [16–18] Also, in relation to change in left atrial diameter under the influence of training in children, several studies, including ours, demonstrated similar results [11,18] The same studies showed no significant changes in left ventricular size. In another study on young athletes, no significant difference was found for aortic dimensions [19] Most discordant observations were related to changes in right ventricular diameter, as some studies reported significant increases associated with sport in children [12,18] and other did not [17]

### The degree of adaptation

An important question arises as to the importance of those changes in clinical practice. The presented results indicate that, despite the fact that mean values of basic cardiac dimensions seem to increase in response to regular training, most of the abnormal results defined as +2 Z-scores do not exceed +2 Z-score values in the general population. Discordance between the two analyzed reference values, in both directions, was found only for a fraction of results (2.4–8.2%). This was not the case for left atrial diameter, where the adaptation to sport seems to be most pronounced and therefore may lead to a greater number of inappropriate indications of an abnormality if one used the general reference values. Also, more than 7% of PWD values in young athletes would be misclassified as abnormal according to Pettersen’s model, whereas they would fit in the normal zone in ours. Surprisingly, for other cardiac dimensions, the situation was the opposite in around 3.9% of cases. However, there is an explanation for this fact.

### How can we explain that?

It may be hypothesized that in children practicing sports there is a lower standard deviation from the mean observed for each dimension. This leads to a situation where, despite significantly higher mean values in this group, the Z-scores are smaller than in the general population. An explanation for that fact could lie in the adaptive process itself. It is possible that the smaller the initial cardiac dimension value (before training), the greater the increase in size (adaptation to training) and vice versa. In other words, the degree of adaptation is not similar in all children, but reversely proportional to initial dimensions. It also suggests that there is an upper limit of adaptation in children, usually not exceeding the normal values found in the general pediatric population. Similar observations were made in relation to cardiac performance indices, where larger increases were found in those who started from lower values, despite similar volume and intensity of training [20].

The difference in the rate of concordant and discordant abnormality indications between LA and IVS or PWD may arise from the fact that the percentage adaptability of these parameters to training is similar (an increase of around 10–15%), but leads to different absolute changes. This translates to only a 1–2 mm increase in the case of LV muscle thickness, but to around 4–5 mm in the case of LA. The latter is therefore more likely to exceed a +2 Z-score on the Pettersen curves, but not our reference values, leading to more discordant results than for IVS and PWD.

### Are specific normative data needed?

All the above considerations lead to a final question: is it necessary to use separate, specific normative data for echocardiographic measurements in children and adolescents practicing sports? We suggest doing so, as the use of the general normative data may occasionally lead to misclassification. However, because of the low rate of discordance in indication of abnormality between the specific and general normative data, the risk of incorrect qualification is relatively low. The finding of a cardiac dimension exceeding reference values in both nomograms should be considered highly suspicious and not related to physiological adaptation to sport.

### Study limitations

Our study has limitations. First, we used 2-dimensional echocardiographic measurements, while the compared study used motion (M) mode views. However, the M-mode measurements of Petterson et al. were guided by 2-dimensional imaging, which is currently the most commonly used and suggested way of cardiac dimension assessment [21]. We have used a Haycock formula to calculate BSA, while in the compared study there is no mention of the method used for BSA calculation, which could have influenced the results. The study subjects were white female and male children and adolescents and therefore presented normative data should be applied only in this group. There has been no information regarding subjects' ethnicity in Pettersen’s study. Ethnic variability, as well as other unconsidered environmental and genetic factors, may influence the comparison. Also, we have studied a mixed group of young athletes, engaged in different sports, which may influence the results in a different way. Nevertheless, majority of training in the young athletes we studied was focused on general development with building of aerobic base and physical activity skills. Therefore, the type, volume and intensity are comparable and general effect on the heart was similar. We must acknowledge that inclusion of participants who underwent echocardiography not routinely, but as a result a suspicion of abnormality could have caused a preselection bias. However, this was a commonly used practice in some [4, 22, 23], but not all [15], of the previous studies on normative data in children. It would be logistically hard and costly to collect such as large group of participants with echocardiography done routinely as it is not a part of pre-participation screening in Poland. Besides it could put some of those children at risk of false positive findings, with all of the consequences described above. Finally, because of the nature of the study, we were unable to analyze young children (under 5 years of age) or in case of adolescents to include maturational state, which could also interfere with the results. This could influence the comparison of results between our group and Pettersen’s group in the lowest ranges of BSA.

## Conclusions

We present normative data for basic echocardiographic measurements in child and adolescent athletes, based on a large sample of children engaged in regular sport training at the local or national level. The cardiac dimensions were higher than in the general pediatric population, particularly left atrial diameter and left ventricular muscle thickness. Nevertheless, in most cases, the upper limit of normality observed in the young athletes was within the limits defined for the general pediatric population. Despite this fact, we suggest using specific normative data for children and adolescents engaged in athletic activities, as some discordant results occurred.

## Supporting information

S1 File Linear dimensions comparison study_database. (CSV)

S2 File Coefficients for regression equations relating echocardiographic measurements and body surface area, mean squared error, and adjusted coefficient of determination which appeared optimal in terms of utilized input variables. (DOCX)
