# Supplementary material for: Comparison of echocardiographic linear dimensions for male and female child and adolescent athletes with published pediatric normative data
Source: PLoS One. 2018 Oct 11;13(10):e0205459. doi: 10.1371/journal.pone.0205459 (PMC6181376; doi:10.1371/journal.pone.0205459)
Supplement: S2 File — (DOCX) [file pone.0205459.s002.docx]

**Table 2.** **Coefficients for regression equations relating echocardiographic measurements and body surface area, mean squared error, and adjusted coefficient of determination which appeared optimal in terms of utilized input variables.**

| **Parameter** | **N** | **Int**  **(β0)** | **p** | **BSA (β1)** | **P** | **BSA^2^**  **(β2)** | **P** | **MSE** | **R^2^** |
| --- | --- | --- | --- | --- | --- | --- | --- | --- | --- |
| LVD | 787 | 0.9517 | <0.001 *** | 0.4514 | <0.001 *** | -0.0536 | <0.001 *** | 0.00349 | 0.732 |
| RVOT PLAX | 788 | - | ns | 0.5373 | <0.001 *** | -0.0501 | <0.001 *** | 0.02752 | 0.941 |
| IVS | 788 | -0.7933 | <0.001 *** | 0.3840 | <0.001 *** | - | Ns | 0.01221 | 0.565 |
| PWD | 782 | -0.7807 | <0.001 *** | 0.3716 | <0.001 *** | - | Ns | 0.00922 | 0.615 |
| LA | 785 | 0.5241 | <0.001 *** | 0.4872 | <0.001 *** | -0.0589 | <0.01 ** | 0.00714 | 0.603 |
| AO | 789 | - | ns | 0.8283 | <0.001 *** | -0.1629 | <0.001 *** | 0.01548 | 0.978 |

AO – aortic sinus diameter at end systole, BSA - body surface area, Int – intercept, IVS – interventricular septum diameter at end diastole, LA – left atrial diameter at end systole, LVD – left ventricular diameter at end diastole, MSE - mean squared error, PWD - left ventricular posterior wall diameter at end diastole, RVOT PLAX - right ventricular diameter in parasternal long axis;

$$Expected y= \beta_{0}+\beta_{1}\cdot BSA+\beta_{2}\cdot{BSA}^{2}+\beta_{3}\cdot{BSA}^{3}$$

$$z=\frac{\ln\left( measured dimension \right)-expected y}{\sqrt{MSE}}$$
